# Azalomycin F_5a_ Eradicates *Staphylococcus aureus* Biofilm by Rapidly Penetrating and Subsequently Inducing Cell Lysis

**DOI:** 10.3390/ijms21030862

**Published:** 2020-01-29

**Authors:** Ganjun Yuan, Pingyi Li, Xuejie Xu, Peibo Li, Qiwang Zhong, Su He, Houqin Yi, Wenfang Yi, Yingying Guan, Zezhang Tom Wen

**Affiliations:** 1 College of Bioscience and Bioengineering, Jiangxi Agricultural University, Nanchang 330045, China; 18270928169@163.com (P.L.); zhongqw2000@163.com (Q.Z.);; 2 Department of Oral and Craniofacial Biology, Louisiana State University Health Sciences Center, New Orleans, LA 70119, USA; 3 Department of Microbiology, Immunology and Parasitology, Louisiana State University Health Sciences Center, New Orleans, LA 70119, USA; 4 School of Life Sciences, Sun Yat-sen University, 135 Xingang Road, Guangzhou 510275, China

**Keywords:** biofilm, azalomycin F, *Staphylococcus aureus*, macrolide, extracellular deoxyribonucleic acid, drop plate, eradication, persister, isolation

## Abstract

Antimicrobial resistance has emerged as a serious threat to public health. Bacterial biofilm, as a natural lifestyle, is a major contributor to resistance to antimicrobials. Azalomycin F_5a_, a natural guanidine-containing polyhydroxy macrolide, has remarkable activities against Gram-positive bacteria, including *Staphylococcus aureus*, a major causative agent of hospital-acquired infections. To further evaluate its potential to be developed as a new antimicrobial agent, its influence on *S. aureus* biofilm formation was evaluated using the crystal violet method, and then its eradication effect against mature biofilms was determined by confocal laser scanning microscopy, the drop plate method, and regrowth experiments. The results showed that azalomycin F_5a_ could significantly inhibit *S. aureus* biofilm formation, and such effects were concentration dependent. In addition, it can also eradicate *S. aureus* mature biofilms with the minimum biofilm eradication concentration of 32.0 μg/mL. As extracellular deoxyribonucleic acid (eDNA) plays important roles in the structural integrity of bacterial biofilm, its influence on the eDNA release in *S. aureus* biofilm was further analyzed using gel electrophoresis. Combined with our previous works, these results indicate that azalomycin F_5a_ could rapidly penetrate biofilm and causes damages to the cell membrane, leading to an increase in DNase release and eventually eradicating *S. aureus* biofilm.

## 1. Introduction

**Figure 1:**
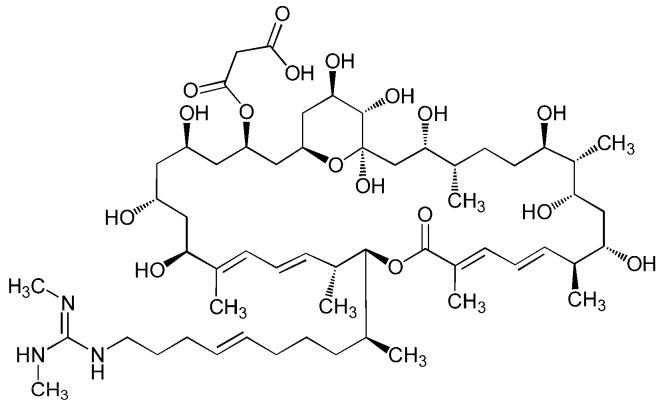
The chemical structure of azalomycin F_5a_.

Azalomycin F_5a_ (Figure 1), produced by marine *Streptomyces* sp. 211726, was a main component of the azalomycin F complex, including twelve 36-membered polyhydroxy macrolides [1,2]. It was also isolated from other streptomycete strains [3,4,5], and has shown remarkable antibacterial and antifungal activities [2,3,4,5]. Simultaneously, many analogs such as guanidylfungins, amycins, shurimycins and niphimycins have been isolated from streptomycete strains [6,7,8,9]. The antimicrobial assays indicated that azalomycin F_5a_, together with its derivatives, had remarkable anti-methicillin-resistant *Staphylococcus aureus* (anti-MRSA) activities [10]. Our recent studies have also shown that azalomycin F_5a_ simultaneously targets cell membrane phospholipid and lipoteichoic acid (LTA), resulting in increases in the cell membrane permeability of *S. aureus* [11]. LTA is an anionic surface polymer anchoring to the cell membrane of Gram-positive bacteria and consisting of glycerol phosphate repeats [12,13]. As LTA plays an essential role in bacterial growth, cell division, biofilm formation, autolysin regulation and resistance to cationic antibiotics [12,13], LTA synthase (LtaS) was proposed as a potential drug target for combating staphylococcal infections [13,14,15]. Thereby, a review on the chemistry, bioactivity and antimicrobial structure–activity relationships of these compounds was recently presented by us [6], and the conclusion is that these compounds have great potential to be developed into antimicrobial drugs.

As antimicrobial resistance is considered a serious threat to human health and economic development, new antimicrobial agents are in desperate need and hot pursuit [16,17]. Many pathogenic bacterial cells can stick to each other on the surfaces of medical devices and other instruments and form complex multi-cellular structures known as biofilms [18,19]. These adherent cells in biofilms are generally embedded within a self-produced matrix, consisting of many extracellular polymeric substances, including polysaccharides and deoxyribonucleic acids (eDNA) [19,20]. As bacterial biofilms can protect cells not only from antimicrobial agents but also from host immune responses [18,19], the biofilm lifestyle can afford bacterial cells a remarkable increase (10 to 1000 folds) in antimicrobial resistance compared to their planktonic counterparts, and probably lead to the bacterial resistance against antimicrobials [19,21,22,23,24]. Simultaneously, *S. aureus* is one of the most frequent causes of biofilm-associated infections among these pathogenic bacteria [18,23,24], and has an inherent ability to form biofilms on various surfaces, including medical devices. Thereby, it is necessary to further evaluate the influence of azalomycin F_5a_, as a representative of these macrolides, on *S. aureus* biofilm.

## 2. Results

### 2.1. Biofilm Formation of S. aureus

**Figure 2:**
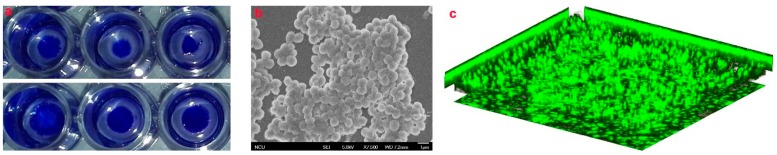
Biofilms of *Staphylococcus aureus*. (**a**) Biofilms, in the wells of two 96-well plates, stained blue with crystal violet; (**b**) biofilms on a plastic disk were presented under scanning electron microscopy (7500×); (**c**) biofilm (634.7 × 634.7 × 54.0 μm^3^, length × width × height) on a silica disk were observed using confocal laser scanning microscopy.

Without the intervention of azalomycin F_5a_, the biofilm of *S. aureus* was formed in the wells of the 96-well plates by following our established protocols [25,26], and the number of biofilms was determined using the crystal violet method. Simultaneously, scanning electron microscopy (SEM) and confocal laser scanning microscopy (CLSM) were used to observe the structure and growth of biofilm covered on disks. The results (Figure 2) showed that the *S. aureus* biofilms were robust under the growth conditions described in Section 4, and could be used for further research.

### 2.2. Influence of Azalomycin F_5a_ on S. aureus Biofilm Formation

**Figure 3:**
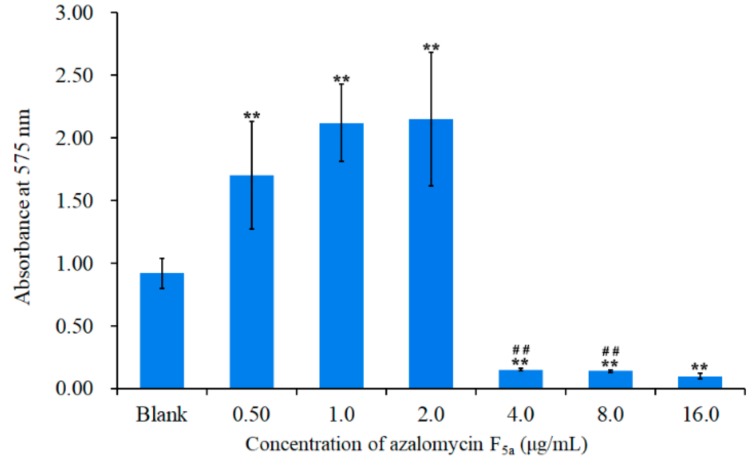
Influence of azalomycin F_5a_ on *S. aureus* biofilm formation. The amount of biofilm with the intervention of various azalomycin F_5a_ concentrations was determined using the crystal violet method; **: *p* < 0.01, means the groups treated with azalomycin F_5a_ showed a significant difference comparing to the blank control; ^##^: *p* < 0.01, means the 4.0 or 8.0 μg/mL group presents a significant difference compared to the 16.0 μg/mL group.

*S. aureus* ATCC 25923 was used as an indicator bacterium for the assessment of azalomycin F_5a_ on bacterial biofilms. The minimum inhibitory concentration (MIC) of azalomycin F_5a_ against this pathogen was determined as 4.0 μg/mL. To evaluate the influence of azalomycin F_5a_ on *S. aureus* biofilm formation, *S. aureus* was grown in TSB supplemented with 1% glucose (TSB-g) in 96-well microtiter plates with and without inclusion of azalomycin F_5a_ at various concentrations. The results are shown in Figure 3, indicating that there was a significant difference between different azalomycin F_5a_ groups and the blank control (*p* < 0.01). Biomass of *S. aureus* biofilm had obviously increased when the concentrations of azalomycin F_5a_ varied from 1/8× to 1/2× that of the MIC, which indicated that azalomycin F_5a_ could promote the growth of *S. aureus* biofilms when its concentration was lower than the MIC. Nevertheless, no significant difference (*p* > 0.05) among the 0.50, 1.0 and 2.0 μg/mL groups was observed. Conversely, biomass of *S. aureus* biofilm had remarkably decreased when the intervention concentrations of azalomycin F_5a_ were greater than or equal to the MIC. This indicated that the minimum biofilm inhibition concentration (MBIC) of azalomycin F_5a_ against *S. aureus* ATCC 25923 is 4.0 μg/mL. Moreover, there were significantly differences (*p* < 0.01) between the 4.0 (or 8.0) and 16.0 μg/mL groups, while no difference (*p* > 0.05) between the 4.0 and 8.0 μg/mL groups were found. In fact, *S. aureus* biofilm was rarely observed in the experiments when the intervention concentration of azalomycin F_5a_ was equal to 16.0 μg/mL.

### 2.3. Influence of Azalomycin F_5a_ on S. aureus Mature Biofilm

**Figure 4:**
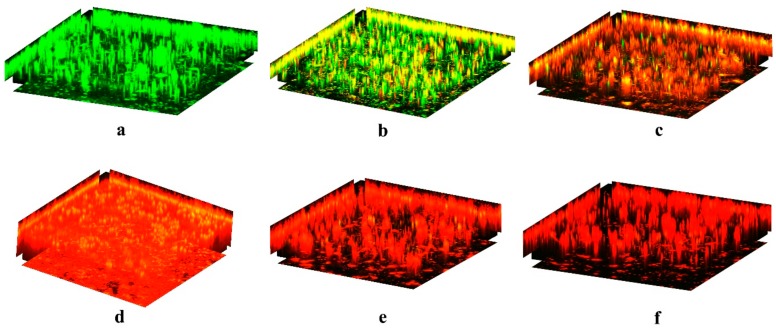
Confocal laser scanning microscopy of *S. aureus* mature biofilms treated with different concentrations of azalomycin F_5a_. (**a**): Blank control; (**b**): 2.0 μg/mL; (**c**): 4.0 μg/mL; (**d**): 8.0 μg/mL; (**e**): 16.0 μg/mL; (**f**): 32.0 μg/mL. Live and dead cells were, respectively, stained in green and red, and yellow meant the superposition of green and red.

**Figure 5:**
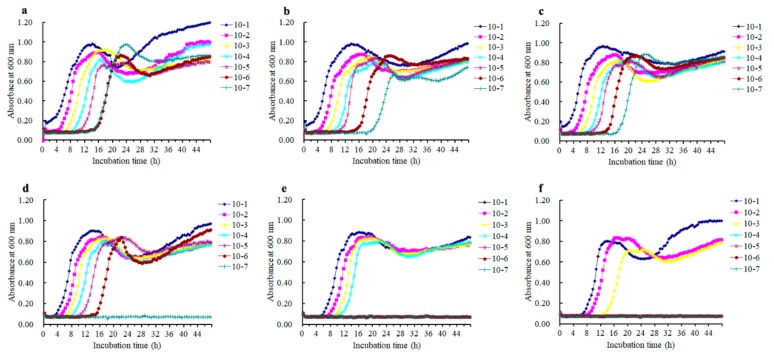
Regrowth of *S. aureus* mature biofilm after being treated with azalomycin F_5a_. (**a**): *S. aureus* mature biofilm; (**b**–**f**): *S. aureus* mature biofilm treated with azalomycin F_5a_ at the concentration of 2.0, 4.0, 8.0, 16.0 and 32.0 μg/mL, respectively.

Biofilms growing on silicone disks were treated with azalomycin F_5a_ at various concentrations, and subsequently the live and dead cells in the remaining biofilms were observed. The results (Figure 4) indicated that azalomycin F_5a_ at concentrations varying from 4.0 to 32.0 μg/mL could obviously kill *S. aureus* cells in biofilm, and that live cells were rarely observed when the concentration of azalomycin F_5a_ increased up to 8× the MIC. Namely, azalomycin F_5a_ could remarkably eradicate *S. aureus* biofilm when its concentration was greater than or equal to 32.0 μg/mL. Moreover, it was worth noting that some cells in *S. aureus* biofilm were killed by azalomycin F_5a_ even though its concentration was less than the MIC (Figure 4b–d), and this indicated that azalomycin F_5a_ could penetrate the biofilm. The above results were also confirmed by the results (Table 1) of counting colony-forming units (CFU), when proper dilutions of the scratched biofilms were plated on brain heart infusion (BHI) agar plates following treatment of the biofilms with azalomycin F_5a_. As shown in Table 1, no visible colonies could be found at the dilution of 10^−4^ or lower levels after biofilm cells were treated with 32.0 μg/mL of azalomycin F_5a_, and even only a rare colony could be observed at the 10^−3^ level. However, many colonies could be observed even at the level of a 10^−7^ dilution after biofilm cells were treated with 2.0 to 16.0 μg/mL of azalomycin F_5a_. These above, by and large, were in accordance with the results (Figure 5) of the regrowth experiments after biofilm cells treated with azalomycin F_5a_ were incubated with TSB medium at 37 °C for 48 h.

### 2.4. The eDNA Content in S. aureus Mature Biofilm after Treated with Azalomycin F_5a_

**Figure 6:**
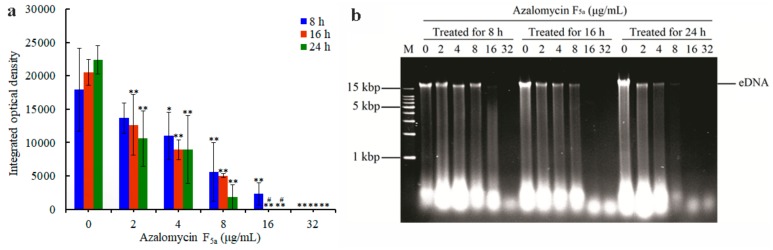
Influence of azalomycin F_5a_ on the eDNA in *S. aureus* mature biofilm. (**a**) Comparing the eDNA content of different azalomycin F_5a_-treated groups with that of the blank control at the same time points, with * or ** indicating significant differences at *p* < 0.05 and *p* < 0.01, respectively. Symbol ^#^ indicates a significant difference (*p* < 0.05) when compared the eDNA contents within the same treatment groups at different time points. (**b**) Representative agarose gel electrophoresis of eDNA in *S. aureus* mature biofilm treated with 2.0, 4.0, 8.0, 16.0 and 32.0 μg/mL of azalomycin F_5a_ for 8, 16 and 24 h, respectively. The optical density of the eDNA band, indicating the relative content of the eDNA, was determined using ImageJ software, and the results were presented as the mean ± SD values of three replicates.

*S. aureus* mature biofilms were treated with azalomycin F_5a_, and the influence of azalomycin F_5a_ on the eDNA in the biofilm is shown in Figure 6a. Compared to the blank control, the eDNA contents of different groups significantly decreased along with the increase in incubation time, while the decreasing rates were different. The higher the treated concentration of azalomycin F_5a_, the faster and more remarkable the reduction of eDNA were. The eDNA contents of the 2.0 μg/mL group for 16 h, and the 4.0, 8.0 and 16.0 μg/mL groups for 8 h were significantly (*p* < 0.05) less than that of the blank control. Even, no eDNA was detected in *S. aureus* mature biofilms after treated with azalomycin F_5a_ at a concentration of 32.0 μg/mL for 8 h and at that of 16.0 μg/mL for 16 h, respectively.

It is worth noting that most of the eDNA (presented mostly in a band of more than 15 kilobase pairs (kbp) in size on the agarose gel) in biofilm treated with azalomycin F_5a_ were degraded (Figure 6b). The lager the treatment concentration of azalomycin F_5a_, the more the degradation of the eDNA contained in the biofilm, and the less the eDNA-degraded substances remaining in the biofilm. For all azalomycin F_5a_ groups, the longer the biofilm was treated with azalomycin F_5a_, the more the eDNA in the biofilms was degraded and the less the eDNA content in the biofilm was. Similarly, the longer the incubation time was, the more the eDNA in the biofilms of blank control were degraded. However, the eDNA content in the biofilm increased along with the increase in incubation time, which was likely attributed to the larger eDNA release than the degradation in the biofilm of the blank controls.

## 3. Discussion

Azalomycin F_5a_, a guanidine-containing polyhydroxy macrolide from some actinomycete strains and has broad-spectrum antimicrobial activities. On one hand, Figure 3 indicated that azalomycin F_5a_ could promote *S. aureus* biofilm formation when its concentration was lower than the MIC. On the other hand, azalomycin F_5a_ could obviously inhibit *S. aureus* biofilm formation at its concentration equal to or greater than the MIC (Figure 3), and its minimum biofilm inhibition concentration (MBIC) was 4.0 μg/mL. The results presented here have shown that azalomycin F_5a_ could significantly inhibit *S. aureus* biofilm formation, and such effects were concentration dependent. Simultaneously, the finding that azalomycin F_5a_ at low concentrations enhances biofilm formation was consistent with previous reports [27,28] that some antibiotics like vancomycin at sub-minimum inhibitory concentrations can enhance biofilm formation. It was thought that antibiotics at concentrations lower than the MIC act as environmental stressors and can generally stimulate the growth of bacterial biofilm [27,28]. This is also in accordance with the fact that biofilm is a way for bacteria to build resistance against harsh and extreme living environments [19]. In order to discover natural products with the potency to eradicate bacterial biofilms, here we mainly focused on the eradicating and inhibitory effect of azalomycin F_5a_ on *S. aureus* biofilm. The molecular mechanism how azalomycin F_5a_ at low concentration promotes *S. aureus* biofilm formation will be explored later. 

Biofilm is a microbial-derived sessile community characterized by cells that are firmly attached to a surface, surrounded by a matrix of an extracellular polymeric substance (EPS) produced by the bacteria themselves [29]. Generally, the biofilm matrix mainly consists of numerous polysaccharides, proteins, eDNA, glycolipids and lipids [29,30,31]. Among them, eDNA, generally released by cell lysis or/and autolysis, plays important roles for bacterial biofilm formation and biofilm structural integrity [32,33,34], and is considered as one of the primary targets for biofilm control and eradicating *S. aureus* biofilm [35,36]. Therefore, the influence of azalomycin F_5a_ on the eDNA in *S. aureus* biofilm was investigated for the probable cause of azalomycin F_5a_ eradicating *S. aureus* biofilm. As shown in Figure 6, the eDNA content in *S. aureus* biofilm following treatment with azalomycin F_5a_ significantly decreased, and such effects were dose-dependent. More eDNA reduction was observed with higher concentrations of azalomycin F_5a_ and a longer time of incubation. As indicated by the eDNA bands and the white spots at the front of the DNA agarose gel (Figure 6b), a large amount of eDNA in the biofilm was deduced to be degraded following treatment with azalomycin F_5a_, especially at higher concentrations (the 8.0, 16.0 and 32.0 μg/mL groups). It is worthy of noting that a substantial amount of the degradation products of the eDNA had likely leaked out of the biofilms, and that is why only trace amounts of eDNA and limited eDNA degradation products could be observed after azalomycin F_5a_ treatment at the concentrations of 8.0, 16.0 and 32.0 μg/mL for 24 h (Figure 6b); this might be due to the formation of smaller nucleic fragments or/and the more serious damage of the biofilm network structure along with an increase in the treating time and azalomycin F_5a_ concentration. In addition, the leakage of eDNA degradation products might be partly driven by the electrostatic effect provided by the positively charged guanidine group of azalomycin F_5a_, since nucleic fragments are negatively charged.

Our previous works indicate that azalomycin F_5a_ could bind to the polar head of cell membrane phospholipids and target lipoteichoic acids, and eventually damage the cell membrane and lead to the cell lysis or/and autolysis of *S. aureus* [11,37]. Therefore, we believe that azalomycin F_5a_ could rapidly penetrate the *S. aureus* biofilm to damage the cell membranes, leading to cell lysis or autolysis, similar to daptomycin being able to quickly penetrate *S. epidermidis* biofilms [38]. This would lead to the rapid release of various enzymes, especially that of DNase, which hydrolyzes eDNA and lead to the degradation of other matrix components in mature biofilm. As eDNA mainly plays a role in the early phase of biofilm formation, DNase treatment for mature biofilm dispersal is no longer substantially effective (Figure 4) [35,39]. However, it can destabilize the interactions between eDNA and other matrix components, and then increase the susceptibility of bacterial cells to antibiotics probably by the permeability increases of the biofilm [35]. These evidences were in accordance with the fact that the higher the concentration of azalomycin F_5a_, the more the DNase release due to cell lysis in the *S. aureus* biofilm, and the faster the eDNA degradation in the biofilm (Figure 6b). All these suggest that the assumption that azalomycin F_5a_ rapidly penetrate biofilm and directly eradicate *S. aureus* biofilm without dispersal is reasonable. This was also confirmed by two following facts: one is that a lot of *S. aureus* cells were directly killed without biofilm dispersal, especially for the concentrations of 16.0 and 32.0 μg/mL (Figure 4e,f); another is that many cells in *S. aureus* biofilm were killed even by the sub-inhibitory concentration of azalomycin F_5a_ (Figure 4b). As biofilm growth is associated with an increased level of mutations, and made bacteria develop a biofilm-specific biocide-resistant phenotype [21,22,23], the characterization of azalomycin F_5a_ rapidly eradicating and penetrating biofilm indicated that it was difficult for *S. aureus* to develop resistance to azalomycin F_5a_.

**Figure 7:**
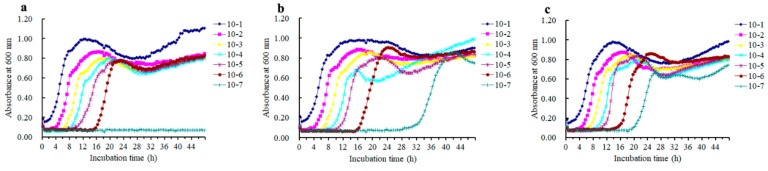
Regrowth of *S. aureus* mature biofilm after treatment with azalomycin F_5a_. (**a**–**c**): *S. aureus* mature biofilms were treated with azalomycin F_5a_ at a concentration of 0.50, 1.0 and 2.0 μg/mL, respectively. It is worth noting that Figure 7c and Figure 5b are the same.

CLSM experiments indicated that the *S. aureus* mature biofilm could be easily eradicated when the concentration of azalomycin F_5a_ increased up to 8× the MIC (Figure 4). So, biofilms treated with azalomycin F_5a_ at concentrations varied from 2.0 to 32.0 μg/mL were also further analyzed by the counting of colony-forming units (CFUs) (Table 1) and regrowth experiments (Figure 5), and the results also confirmed that azalomycin F_5a_ could remarkably eradicate *S. aureus* biofilm. Unexpectedly, the CFU of the 2.0 μg/mL azalomycin F_5a_ group was significantly less than that of the 4.0 μg/mL group (Table 1). In fact, this was also observed from the regrowth experiments (Figure 5b,c). From Figure 5b (2.0 μg/mL group), the biofilm cells with a 10^−7^ dilution regrew after incubation at 37°C for 20 h, while those of the 4.0 μg/mL group regrew after incubation at 37°C for 17 h. Simultaneously, the absorbance of wells in the 2.0 μg/mL group was 0.40 to 0.50 after 24 h incubation, while those in the 1× MIC group was approximately up to 0.85. To elucidate the probable underlying factors, the regrowth of *S. aureus* mature biofilm treated with 1.0 and 0.50 μg/mL of azalomycin F_5a_ was also determined by us. The results indicated that the lower the concentration (less than MIC) of azalomycin F_5a_, the longer it took for live cells in the 10^−7^ dilution to regrow (Figure 7). Even more unexpectedly, no cells regrew in the 10^−7^ dilution of the 0.50 μg/mL group during the regrowth experiments after 48 h. To the best of our knowledge, this phenomenon is the first to be reported. The underlying mechanisms, however, await further investigation. Bacteria, especially in biofilms, can develop persistent cells that are highly resistant to host immune attack, various environment stress and antibiotics [40,41,42,43,44]. Based on the results of the regrowth experiment of cells in the 10^−7^ dilution of the 2.0, 1.0 and 0.50 μg/mL groups (Figure 7), we deduced that the lower the concentration of azalomycin F_5a_ used to treat *S. aureus* biofilms, the greater the proportion of persistent cells there would be. If that is the case, this would also provide a good method for isolating the bacterial persisters.

Generally speaking, the pour, spread and drop plate techniques can be used for the enumeration of colony-forming units (CFUs), and there are no significant differences among these three plating methods [45]. However, the drop plate method has many advantages, being convenient to use, economical and less time consuming [45,46], and is especially suitable for the comparison of large experimental groups. Therefore, the drop plate method was selected for counting CFUs, referring to previous publications [46,47]. To reduce the random error, five 10 μL-drops from each well were plated on a TSA medium, and incubated at 37 °C for 24 h. The results, by and large, were consistent with the regrowth experiments. 

In a previous publication [48], the MBEC was defined as the minimal concentration of antibiotic reducing biofilm cells below the detection limit of the assays used, such as 10^2^ CFU/mL for the cell counting method. However, the MBEC values could not be determined using a resazurin-based assay or the cell counting method in this paper, and only the percentage of decrease compared to untreated samples was calculated at the upper limit of the measured concentration. In their experiments, the results indicated that the resazurin-based assay had some limitation as what Peeters et al. reported [49]. Simultaneously, the log_10_ CFU reduction was presented as the results of the cell counting method. As the initial biofilm biomass would greatly influence the results of the cell counting method in different experiments, the log_10_ CFU reduction should be more reliable and scientific than the cell number to present the practical effect of the antimicrobial agents on the biofilm cells. Table 1 suggested that a 5 log_10_ CFU reduction, compared to the blank groups, was presented on *S. aureus* mature biofilm after treatment with 32.0 ug/mL of azalomycin F_5a_. This result was also confirmed by the regrowth experiments for 48 h (Figure 5f presented at least a 4 log_10_ CFU reduction compared to Figure 5a) and the CLSM results, where almost all the cells in the biofilm were killed (Figure 4f). Thereby, the live cell number in *S. aureus* mature biofilm treated with 32.0 ug/mL of azalomycin F_5a_ presented a 4 to 5 log_10_ CFU reduction compared to those in the blank groups.

Considering the complex of antimicrobial agents treating infection and preventing antimicrobial resistance in vivo [50], it is impracticable and unnecessary to completely eradicate biofilm. So, a 4 to 5 log_10_ CFU reduction of live cells in biofilm, which indicates the killing of at least 99.99% cells in *S. aureus* mature biofilm, should be considered as a complete biofilm eradication. Based on this, the minimal biofilm eradication concentration (MBEC) of azalomycin F_5a_ against *S. aureus* biofilm is 32.0 μg/mL.

## 4. Materials and Methods 

### 4.1. Azalomycin F_5a_

Azalomycin F_5a_ (purity, 98.2%) was isolated from the fermentation of *Streptomyces hygroscopicus* var. *azalomyceticus* according to our published methods [1], and widely used in our previous works [11,37,51]. The stock solution of azalomycin F_5a_, stored at −20 °C, was prepared by dissolving in dimethyl sulfoxide (DMSO) to obtain a concentration of 2048 μg/mL. The stock solution was diluted to the desired concentrations with tryptic soy broth (TSB) (Haibo Biotechnology Co., Ltd., Qingdao, China) immediately before use. In another, the DMSO concentrations in all the test systems were kept to less than or equal to 1.56%, and all those in the blank controls or 0× MIC azalomycin F_5a_ groups were 1.56%.

### 4.2. Bacterial Strains and Growth Condition

*S. aureus* ATCC 25923 was purchased from American Type Culture Collection, Manassas, VA, USA. This organism was stored as frozen stocks at −80 °C. Prior to use, *S. aureus* was cultured onto trypticase soy agar (TSA) (Haibo Biotechnology Co., Ltd., Qingdao, China) plate at 37 °C, and then pure colonies from the plate were inoculated into TSB at 37 °C for 24 h on a rotary shaker (160 rpm). A 1:100 dilution of the overnight culture was made into fresh TSB, and then incubated at 37 °C until the exponential phase for the following experiments. TSB and TSB supplemented with 0.5% glucose (TSB-g) were respectively used for the antimicrobial susceptibility and the biofilm-related tests. 

### 4.3. Antimicrobial Susceptibility Assay

According to the standard procedure described by the Clinical and Laboratory Standards Institute (CLSI) [52], the exponential phase culture was diluted with TSB to achieve an *S. aureus* concentration ≈ 1.0×10^6^ CFU/mL, and then the susceptibility of azalomycin F_5a_ against *S. aureus* ATCC 25923 was determined using the broth microdilution method on 96-well plates in triplicate [50]. The minimum inhibitory concentration (MIC) was defined as the lowest concentration of azalomycin F_5a_ that completely inhibited bacterial growth as detected by the unaided eye when the growth of *S. aureus* in the blank wells was good.

### 4.4. Biofilm Formation

Referring to previous reports [26,53,54,55], the exponential phase culture was diluted with TSB-g to achieve the *S. aureus* concentration of approximately 5×10^7^ CFU/mL, and then 200 μL of diluted *S. aureus* culture was added into each well of a 96-well microtiter plate. The plates were incubated at 37 °C for 24 h to induce biofilm formation. For scanning electron microscopy (SEM) and confocal laser scanning microscopy (CLSM) experiments, the experimental setup was the same as that described above, except that each well erectly contained a sterilized silicone disk or a sterilized plastic disk. Control wells contained TSB-g alone, or TSB-g and *S. aureus*.

### 4.5. Biofilm Formation Assay

Biofilm formation was carried out on 96-well microtiter plates according to the same procedure as Section 4.4 (Biofilm Formation), except that the 200 μL microbial suspensions, respectively, contained azalomycin F_5a_ with various concentrations of 0, 0.5, 1.0, 2.0, 4.0, 8.0 and 16.0 μg/mL (equal to 0×, 1/8×, 1/4×, 1/2×, 1×, 2× and 4× the MIC of azalomycin F_5a_ against *S. aureus*) in TSB-g. To quantify the biofilm, 0.1% crystal violet staining was used as previously described [26] with a little modification. Briefly, the planktonic cultures were gently removed from the wells of the 96-well plates after incubation at 37 °C for 24 h. Then, the wells were washed twice with water, air-dried, stained with 0.1% crystal violet solution for 15 min and repeatedly washed with water to remove excess dye. The stained cells were resolubilized in a 200 μL mixture of ethanol–acetone (80:20), and the absorbance of the crystal violet was determined at 575 nm on a TECAN Infinite 200 Pro microplate reader (Tecan Austria GmbH, Grödig, Austria). The experiment was performed in triplicate. 

### 4.6. Biofilm Eradication Assay

Biofilms were grown on silicone disks as described above. The disks were taken out and gently washed twice with phosphate-buffered saline (PBS) to remove nonadherent cells, and then put into another 96-well plate that, respectively, contained azalomycin F_5a_ at various concentrations of 0, 2.0, 4.0, 8.0, 16.0 and 32.0 μg/mL (equal to 0×, 1/2×, 1×, 2×, 4× and 8× of the MIC of azalomycin F_5a_ against *S. aureus*) in TSB-g. After the plate was incubated at 37 °C for 24 h, the disks were taken out from the wells, and gently washed twice with PBS. For the same group, the remaining biofilms on two disks were observed using CLSM for live and dead cells [25,26,53], those on three disks were used for counting colony-forming units and those on another three disks were used for regrowth experiments.

### 4.7. Scanning Electron Microscopy

Biofilms without the treatment of azalomycin F_5a_ were grown on plastic disks as described above. Referring to the method reported by Peters et al. [26], samples were first washed three times with PBS to remove planktonic cells, and then placed into 2.5% glutaraldehyde for 24 h at 4 °C. Secondly, the disks were gradually dehydrated in a series of ethanol solutions (25%, 50%, 75%, 90%, 95% and 100%) with a 10 min interval, and subsequently washed with hexamethyldisilazane for 20 min and desiccated to achieve complete dehydration. Finally, samples were mounted on aluminum stubs with double-sided carbon tape and sputter coated with carbon. To assess the biofilm structure, Biofilms on disks were imaged with a JSM-6701F scanning electron microscope (JEOL, Tokyo, Japan) with the voltage set to 5.0 kV. 

### 4.8. Confocal Laser Scanning Microscopy

Biofilms were grown on silicone disks as described above. After treated with azalomcyin F_5a_, the discs were gently washed twice with PBS to remove nonadherent cells, and then erectly placed in the wells of a 24-well plate containing PBS and nucleic acid stains SYTO-9/PI (1:5) provided in a BacLight Live/Dead staining kit (Invitrogen) [11,25,26]. After the plate stayed for 15 min at 25 °C in the dark, the disks were taken from wells, and observed under a confocal laser scanning microscope (Olympus Fluoview^TM^ FV1000) equipped with a detector and filter sets for monitoring SYTO-9 and PI. 

### 4.9. Drop Plate for Counting Colony-Forming Units

**Figure 8:**
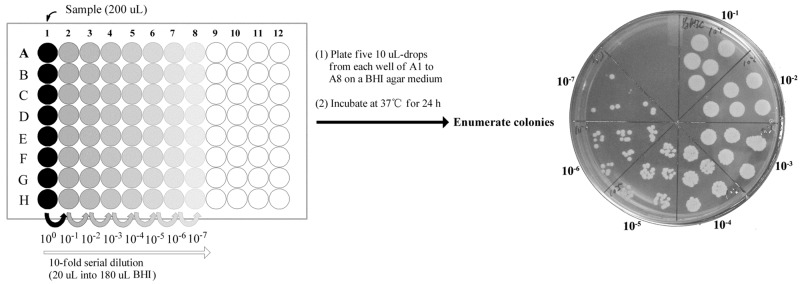
Scheme of the drop plate assay for counting colony-forming units.

Following a previously published protocol [26,46,47], the drop plate method was used for enumeration of CFUs with a little modification. After the biofilms that were grown on the silicone disks (as described above) were treated with azalomycin F_5a_, the disks with adherent biofilm were aseptically transferred to a new 96-well plate, in which each well contained 200 μL PBS, and gently sonicated in a DK-410T water bath sonicator with a frequency of 40 kHz for 5 min to dislodge biofilm-embedded *S. aureus*. As shown on Figure 8, serial decimal dilutions were made in the sterile BHI broth, and five 10 μL-drops from each well were placed onto a section of the BHI agar plate. Following incubation at 37 °C for 24 h, colonies were counted and expressed as the number of CFUs/50 μL (mean ± SD, *n* = 3).

### 4.10. Regrowth Experiment

Biofilms were grown on silicone disks as described above. After treatment with azalomycin F_5a_ at 37 °C for 24 h, regrowth of *S. aureus* in biofilms was tested. Briefly, the disks were gently washed with PBS twice, and then were aseptically transferred to the wells (row 1) of a 96-well plate of which each well contained 150 μL TSB. After gently sonicated in a DK-410T water bath sonicator with a frequency of 40 kHz for 5 min to dislodge biofilm-embedded *S. aureus*, the suspension and following 150 μL TSB washing liquid of each disk were transferred into the corresponding well of a 100-well plate well, and the well mixed to obtain a 300 μL bacterial suspension. Then, serial decimal dilutions with TSB were made on the 100-well plate to obtain bacterial dilutions from the 10^0^ to around 10^−7^ levels, and the plate then incubated at 37 °C for 48 h. The regrowth was monitored with a Bioscreen C (Oy Growth Curves AB Ltd., Helsinki, Finland), and the optical density was recorded at 600 nm with an interval of 30 min. 

### 4.11. Influence of Azalomycin F_5a_ on the eDNA in S. aureus Mature Biofilm

Biofilm formation was carried out on 48-well microtiter plates according to the same procedure as Section 4.4 (Biofilm Formation). After gently removing planktonic culture, 1.0 mL TSB-g medium, respectively containing azalomycin F_5a_ at various concentrations of 0, 2.0, 4.0, 8.0, 16.0 and 32.0 μg/mL, was gently added into the corresponding wells, and then cultured at 37 °C for 8, 16 and 24 h, respectively. Next, eDNA in the *S. aureus* biofilm was extracted and purified by a previous method with some modifications [56,57]. Briefly, the planktonic culture was discarded, and the biofilm adhering to the wells was washed twice with PBS. Then, the biofilm was removed from the surface of the wells using a plastic tissue culture cell scraper after adding 100 μL TES (10 mM Tris-HCl, 10 mM EDTA, 500 mM NaCl, pH = 8.0) at 4 °C. After being mixed five times for 30 s each time on a vortex device, the mixture was centrifuged at 12,000× *g* for 30 min to acquire a supernatant containing eDNA. Then, the supernatant was extracted with an equal volume of phenol:chloroform:isoamyl alcohol (25:24:1, *v*/*v*) and chloroform:isoamyl alcohol (24:1, *v*/*v*) in turn. The aqueous phase was obtained by centrifugation at 12,000× *g* for 10 min, and to which triple volumes of cold ethanol and 1/10 volume of 3.0 mol/L sodium acetate (cold, pH = 5.2) were added. It was then allowed to incubate overnight at −20 °C to precipitate eDNA. The pellet containing eDNA was obtained by centrifugation at 12,000× *g* for 30 min, washed with 75% cold ethanol and air dried. After the pellet was resuspended in 15 μL TE buffer (10 mM Tris-HCl, 1.0 mM EDTA, pH = 8.0), the eDNA was analyzed by 1.0% agarose gel electrophoresis, and visualized using an Automatic Gel Imaging Analysis System (Peiqing Science & Technology Co., Ltd., Shanghai, China). Utilizing open-source ImageJ analysis software (U.S. National Institutes of Health, Bethesda, MD, USA.) [58,59], the quantification of eDNA was achieved. The experiment was performed in triplicate.

### 4.12. Statistics

Biomasses from crystal violet staining and the CFU counts from the silicone disks were compared using a Student’s t test, as well as one- or two-way analyses of variance (ANOVAs) with Duncan’s method used for multiple comparisons. The different symbols indicated significant difference among different treatments at a *p* value of < 0.05 or 0.01. All statistical analyses were performed in and the graphs composed with Data Processing System (version 7.05) (Hangzhou RuiFeng Information Technology Co., Ltd., Hangzhou, China), Microsoft Excel software (Microsoft, Redmond, WA, USA) and/or Origin 8.5 (OriginLab Corporation, Northampton, MA, USA). 

## 5. Conclusions

Azalomycin F_5a_, a representative compound of guanidine-containing polyhydroxy macrolides, can remarkably inhibit *S. aureus* biofilm formation, and such effects were concentration dependent. Furthermore, azalomycin F_5a_ can eradicate *S. aureus* mature biofilm with an MBEC of 32.0 μg/mL. Combined with our previous works, the results presented here have further indicated that azalomycin F_5a_ could rapidly penetrate *S. aureus* biofilm and damage the cell membranes, leading to an increase in DNase release by inducing cell lysis or/and autolysis, and eventually eradicating *S. aureus* mature biofilms.

## Figures and Tables

**Table 1:** Drop plate for counting colony-forming units (mean ± SD, *n* = 3).

Sample ^1^	CFUs/50 μL						
	10^−^^1^	10^−^^2^	10^−^^3^	10^−^^4^	10^−^^5^	10^−^^6^	10^−^^7^
Model	UC ^2^	UC	UC	UC	UC	37 ± 6	37 ± 7
Model + Blank	UC	UC	UC	UC	UC	60 ± 8	39 ± 6
Model + 2.0 μg/mL	UC	UC	UC	UC	60 ± 6	35 ± 7	5 ± 2
Model + 4.0 μg/mL	UC	UC	UC	UC	59 ± 12	31 ± 6	22 ± 7
Model + 8.0 μg/mL	UC	UC	UC	UC	30 ± 5	23 ± 5	12 ± 3
Model + 16.0 μg/mL	UC	UC	UC	25 ± 6	3 ± 1	3 ± 2	2 ± 2
Model + 32.0 μg/mL	UC	37 ± 9	3 ± 2	0	0	0	0

^1^ Model, *S. aureus* mature biofilm without further incubation in 200 μL TSB and treatment of azalomycin F_5a_; Model + Blank, *S. aureus* mature biofilm further incubated in 200 μL TSB while no treatment of azalomycin F_5a_; Model + 2.0, 4.0, 8.0, 16.0 and 32.0 μg/mL, *S. aureus* mature biofilm further treated with azalomycin F_5a_ at the concentration of 2.0, 4.0, 8.0, 16.0 and 32.0 μg/mL, respectively.^2^ UC, Uncounted as there were too many to count accurately.

## Abbreviations

MIC	Minimum Inhibitory Concentration
MBEC	Minimum Biofilm Eradication Concentration
MBIC	Minimum Biofilm Inhibition Concentration
SEM	Scanning Electron Microscopy
CLSM	Confocal Laser Scanning Microscopy
eDNA	Extracellular Deoxyribonucleic Acid
DNase	Deoxyribonuclease
LTA	Lipoteichoic Acid
LtaS	Lipoteichoic Acid Synthase
CFU	Colony-Forming Unit
TSB	Trypticase Soy Broth

## References

[1] Yuan G., Lin H., Wang C., Hong K., Liu Y., Li J. (2011). ^1^H and ^13^C assignments of two new macrocyclic lactones isolated from *Streptomyces* sp. 211726 and revised assignments of azalomycins F_3a_, F_4a_ and F_5a_. Magn. Reson. Chem..

[2] Yuan G., Hong K., Lin H., She Z., Li J. (2013). New azalomycin F analogs from mangrove *Streptomyces* sp. 211726 with activity against microbes and cancer cells. Mar. Drugs.

[3] Iwasaki S., Namikoshi M., Sasaki K., Fukushima K., Okuda S. (1982). Studies on macrocyclic lactone antibotics.V.1) The structures of azalomycins F_3a_ and F_5a_. Chem. Pharm. Bull..

[4] Chandra A., Nair M.G. (1995). Azalomycin F complex from *Streptomyces hygroscopicus*, MSU/MN-4-75B. J. Antibiot..

[5] Cheng J., Yang S.H., Palaniyandi S.A., Han J.S., Yoon T., Kim T., Suh J. (2010). Azalomycin F complex is an antifungal substance produced by *Streptomyces malaysiensis* MJM1968 isolated from agricultural soil. J. Korean Soc. Appl. Biol. Chem..

[6] Song X., Yuan G., Li P., Cao S. (2019). Guanidine-containing polyhydroxyl macrolides: Chemistry, biology, and structure-activity relationship. Molecules.

[7] Takesako K., Beppu T. (1984). Studies on new antifungal antibiotics, guanidylfungins A and B. I. Taxonomy, fermentation, isolation and characterization. J. Antibiot..

[8] Grabley S., Hammann P., Raether W., Wink J., Zeeck A. (1990). Secondary metabolites by chemical screening: II. Amycins A and B two novel niphimycin analogs isolated from a high producer strain of elaiophylin and nigericin. J. Antibiot..

[9] Hu Y., Wang M., Wu C., Tan Y., Li J., Hao X., Duan Y., Guan Y., Shang X., Wang Y. (2018). Identification and proposed relative and absolute configurations of niphimycins C-E from the marine-derived *Streptomyces* sp. IMB7-145 by genomic analysis. J. Nat. Prod..

[10] Yuan G., Li P., Yang J., Pang H., Pei Y. (2014). Anti-methicillin-resistant *Staphylococcus aureus* assay of azalomycin F_5a_ and its derivatives. Chin. J. Nat. Med..

[11] Yuan G., Xu L., Xu X., Li P., Zhong Q., Xia H., Hu Y., Li P., Song X., Li J. (2019). Azalomycin F_5a_, a polyhydroxy macrolide binding to the polar head of phospholipid and targeting to lipoteichoic acid to kill methicillin-resistant *Staphylococcus aureus*. Biomed. Pharmacother..

[12] Xia G., Kohler T., Peschel A. (2010). The wall teichoic acid and lipoteichoic acid polymers of *Staphylococcus aureus*. Int. J. Med. Microbiol..

[13] Vickery C.R., Wood B.M., Morris H.G., Losick R., Walker S. (2018). Reconstitution of *Staphylococcus aureus* lipoteichoic acid synthase activity identifies Congo red as a selective inhibitor. J. Am. Chem. Soc..

[14] Richter S.G., Elli D., Kim H.K., Hendrickx A.P.A., Sorg J.A., Schneewind O., Missiakas D. (2013). Small molecule inhibitor of lipoteichoic acid synthesis is an antibiotic for Gram-positive bacteria. Proc. Nat. Acad. Sci. USA.

[15] Percy M.G., Gründling A. (2014). Lipoteichoic acid synthesis and function in gram-positive bacteria. Annu. Rev. Microbiol..

[16] Laxminarayan R., Sridhar D., Blaser M., Wang M., Woolhouse M. (2016). Achieving global targets for antimicrobial resistance. Science.

[17] Tacconelli E., Sifakis F., Harbarth S., Schrijver R., van Mourik M., Voss A., Sharland M., Rajendran N.B., Rodríguez-Baño J., Bielicki J. (2018). Surveillance for control of antimicrobial resistance. Lancet. Infect. Dis..

[18] Bjarnsholt T., Ciofu O., Molin S., Givskov M., Høiby N. (2013). Applying insights from biofilm biology to drug development - can a new approach be developed?. Nat. Rev. Drug. Discov..

[19] Kostakioti M., Hadjifrangiskou M., Hultgren S.J. (2013). Bacterial biofilms: Development, dispersal, and therapeutic strategies in the dawn of the postantibiotic era. Cold. Spring Harb. Perspect. Med..

[20] Flemming H., Wingender J. (2010). The biofilm matrix. Nat. Rev. Microbiol..

[21] Hoiby N., Bjarnsholt T., Givskov M., Molin S., Ciofu O. (2010). Antibiotic resistance of bacterial biofilms. Int. J. Antimicrob. Agents.

[22] Mah T.F., O’Toole G.A. (2001). Mechanisms of biofilm resistance to antimicrobial agents. Trends Microbiol..

[23] Stewart P.S., Costerton J.W. (2001). Antibiotic resistance of bacteria in biofilms. Lancet.

[24] Antunes A.L., Bonfanti J.W., Perez L.R., Pinto C.C., Freitas A.L., Macedo A.J., Barth A.L. (2011). High vancomycin resistance among biofilms produced by *Staphylococcus* species isolated from central venous catheters. Mem. Inst. Oswaldo Cruz.

[25] Bitoun J.P., Nguyen A.H., Fan Y., Burne R.A., Wen Z.T. (2011). Transcriptional repressor Rex is involved in regulation of oxidative stress response and biofilm formation by *Streptococcus mutans*. FEMS Microbiol. Lett..

[26] Peters B.M., Ward R.M., Rane H.S., Lee S.A., Noverr M.C. (2013). Efficacy of ethanol against *Candida albicans* and *Staphylococcus aureus* polymicrobial biofilms. Antimicrob. Agents Chemother..

[27] Chen H.Y., Chen C.C., Fang C.S., Hsieh Y.T., Lin M.H., Shu J.C. (2011). Vancomycin activates σ^B^ in vancomycin-resistant *Staphylococcus aureus* resulting in the enhancement of cytotoxicity. PLoS ONE.

[28] Hsu C.Y., Lin M.H., Chen C.C., Chien S.C., Cheng Y.H., Su I.N., Shu J.C. (2011). Vancomycin promotes the bacterial autolysis, release of extracellular DNA, and biofilm formation in vancomycin-non-susceptible *Staphylococcus aureus*. FEMS Immunol. Med. Microbiol..

[29] Donlan R.M. (2002). Biofilms: Microbial life on surfaces. Emerg. Infect. Dis..

[30] McCrate O.A., Zhou X., Reichhardt C., Cegelski L. (2013). Sum of the parts: Composition and architecture of the bacterial extracellular matrix. J. Mol. Biol..

[31] Donlan R.M., Costerton J.W. (2002). Biofilms: Survival mechanisms of clinically relevant microorganisms. Clin. Microbiol. Rev..

[32] Okshevsky M., Meyer R.L. (2013). The role of extracellular DNA in the establishment, maintenance and pertubation of bacterial biofilms. Crit. Rev. Microbiol..

[33] Jakubovics N.S., Shields R.C., Rajarajan N., Burgess J.G. (2013). Life after death: The critical role of extracellular DNA in microbial biofilms. Lett. Appl. Microbiol..

[34] Das T., Sehar S., Manefield M. (2013). The roles of extracellular DNA in the structural integrity of extracellular polymeric substance and bacterial biofilm development. Environ. Microbiol. Rep..

[35] Okshevsky M., Regina V.R., Meyer R.L. (2015). Extracellular DNA as a target for biofilm control. Curr. Opin. Biotechnol..

[36] Sugimoto S., Sato F., Miyakawa R., Chiba A., Onodera S., Hori S., Mizunoe Y. (2018). Broad impact of extracellular DNA on biofilm formation by clinically isolated methicillin-resistant and -sensitive strains of *Staphylococcus aureus*. Sci. Rep..

[37] Xu L., Xu X., Yuan G., Wang Y., Qu Y., Liu E. (2018). Mechanism of azalomycin F_5a_ against methicillin-resistant *Staphylococcus aureus*. Biomed. Res. Int..

[38] Stewart P.S., Davison W.M., Steenbergen J.N. (2009). Daptomycin rapidly penetrates a *Staphylococcus epidermidis* biofilm. Antimicrob. Agents Chemother..

[39] Castillo Pedraza M.C., Novais T.F., Faustoferri R.C., Quivey R.G., Terekhov A., Hamaker B.R., Klein M.I. (2017). Extracellular DNA and lipoteichoic acids interact with exopolysaccharides in the extracellular matrix of *Streptococcus mutans* biofilms. Biofouling.

[40] Lewis K. (2010). Persister cells. Annu. Rev. Microbiol..

[41] Gollan B., Grabe G., Michaux C., Helaine S. (2019). Bacterial persisters and infection: Past, present, and progressing (Review). Annu. Rev. Microbiol..

[42] Lee J.H., Kim Y.G., Gwon G., Wood T.K., Lee J. (2016). Halogenated indoles eradicate bacterial persister cells and biofilms. AMB Express.

[43] Podlesek Z., Butala M., Šakanović A., Žgur-Bertok D. (2016). Antibiotic induced bacterial lysis provides a reservoir of persisters. Antonie van Leeuwenhoek.

[44] Maisonneuve E., Gerdes K. (2014). Molecular mechanisms underlying bacterial persisters. Cell.

[45] Hoben H.J., Somasegaran P. (1982). Comparison of the pour, spread, and drop plate methods for enumeration of *Rhizobium* spp. in inoculants made from presterilized peatt. Appl. Environ. Microb..

[46] Chen C., Nace G.W., Irwin P.L. (2003). A 6×6 drop plate method for simultaneous colony counting and MPN enumeration of *Campylobacter jejuni*, *Listeria monocytogenes*, and *Escherichia coli*. J. Microbiol. Meth..

[47] Herigstad B., Hamilton M., Heersink J. (2001). How to optimize the drop plate method for enumerating bacteria. J. Microbiol. Meth..

[48] Cruz C.D., Shah S., Tammela P. (2018). Defining conditions for biofilm inhibition and eradication assays for Gram-positive clinical reference strains. BMC Microbiol..

[49] Peeters E., Nelis H.J., Coenye T. (2008). Comparison of multiple methods for quantification of microbial biofilms grown in microtiter plates. J. Microbiol. Methods.

[50] Xu X., Xu L., Yuan G., Wang Y., Qu Y., Zhou M. (2018). Synergistic combination of two antimicrobial agents closing each other’s mutant selection windows to prevent antimicrobial resistance. Sci. Rep..

[51] Xu X., Wu X., Yuan G., Xu L., Wang Y. (2016). Mutant selection windows of azalomycin F_5a_ in combination with vitamin K_3_ against methicillin-resistant *Staphylococcus aureus*. J. Biosci. Med..

[52] Clinical and Laboratory and Standards Institute (CLSI) (2015). Methods for Dilution Antimicrobial Susceptibility Tests for Bacteria That Grow Aerobically.

[53] Lin Y.J., Alsad L., Vogel F., Koppar S., Nevarez L., Auguste F., Seymour J., Syed A., Christoph K., Loomis J.S. (2013). Interactions between *Candida albicans* and *Staphylococcus aureus* within mixed species biofilms. BIOS.

[54] Hou Y., Wang Z., Zhang P., Bai H., Sun Y., Duan J., Mu H. (2017). Lysozyme associated liposomal gentamicin inhibits bacterial biofilm. Int. J. Mol. Sci..

[55] Kim M.K., Kang N.H., Ko S.J., Park J., Park E., Shin D.W., Kim S.H., Lee S.A., Lee J.I., Lee S.H. (2018). Antibacterial and antibiofilm activity and mode of action of magainin 2 against drug-resistant *Acinetobacter baumannii*. Int. J. Mol. Sci..

[56] Shields R.C., Mokhtar N., Ford M., Hall M.J., Burgess J.G., ElBadawey M.R., Jakubovics N.S. (2013). Efficacy of a marine bacterial nuclease against biofilm forming microorganisms isolated from chronic rhinosinusitis. PLoS ONE.

[57] Kreth J., Vu H., Zhang Y., Herzberg M.C. (2009). Characterization of hydrogen peroxide-induced DNA release by *Streptococcus sanguinis* and *Streptococcus gordonii*. J. Bacteriol..

[58] Rasband W.S. (2018). ImageJ.

[59] Craig J.M., Vena N., Ramkissoon S., Idbaih A., Fouse S.D., Ozek M., Sav A., Hill D.A., Margraf L.R., Eberhart C.G. (2012). DNA fragmentation simulation method (FSM) and fragment size matching improve aCGH performance of FFPE tissues. PLoS ONE.

